# In Vitro Evaluation of Fosfomycin Combinations Against Metallo-β-Lactamase-Producing *Klebsiella pneumoniae* and *Pseudomonas aeruginosa* Clinical Isolates

**DOI:** 10.3390/antibiotics14121247

**Published:** 2025-12-10

**Authors:** Esther Wohlfarth, Aurélien Dinh, Georgia Vrioni, Dorota Żabicka, Mariano Bernardo, Carlo Tascini, Latifa Noussair, Christian Mayer

**Affiliations:** 1 Antiinfectives Intelligence GmbH, 51105 Cologne, Germany; 2 Infectious Disease Department, University Hospital Raymond-Poincaré, 92380 Garches, France; aurelien.dinh@aphp.fr; 3 Department of Microbiology, Medical School, National and Kapodistrian University of Athens, 11527 Athens, Greece; gvrioni@med.uoa.gr; 4 Department of Epidemiology and Clinical Microbiology, National Medicines Institute, 00725 Warsaw, Poland; d.zabicka@nil.gov.pl; 5 Microbiology and Virology Unit, Cotugno Hospital, Azienda Ospedaliera dei Colli, 80131 Naples, Italy; mariano.bernardo@ospedalideicolli.it; 6 Department of Medicine (DMED), Infectious Diseases Clinic, University of Udine, 56100 Udine, Italy; c.tascini@gmail.com; 7 Microbiology Department, University Hospital Raymond-Poincaré, 92380 Garches, France; latifa.noussair@aphp.fr; 8 InfectoPharm Arzneimittel und Consilium GmbH, 64646 Heppenheim, Germany; christian.mayer@infectopharm.com

**Keywords:** fosfomycin, ceftazidime–avibactam, antimicrobial resistance (AMR), carbapenem resistance, multidrug resistance (MDR), synergy, metallo-β-lactamase (MBL), *Klebsiella pneumoniae*, *Pseudomonas aeruginosa*, cefiderocol, fractional inhibitory concentration (FIC)

## Abstract

**Background/Objectives**: Metallo-β-lactamase (MBL)-producing Gram-negative bacteria represent a growing global health threat due to their broad resistance to β-lactam antibiotics, including carbapenems, which severely limits treatment options. This study aimed to evaluate the in vitro synergistic activity of fosfomycin (FOS) in combination with selected older and newer antimicrobials against MBL-producing *Klebsiella pneumoniae* and *Pseudomonas aeruginosa*. **Methods**: Synergistic interactions were assessed using agar dilution checkerboard on 42 MBL-producing clinical isolates (22 *K. pneumoniae*, 20 *P. aeruginosa*) and confirmed using time-kill assays with selected isolates. FOS was tested in combination with colistin (COL), ceftazidime–avibactam (CAZ-AVI), meropenem (MER), amikacin (AMI), aztreonam (AZT), aztreonam–avibactam (AZT-AVI), or cefiderocol (FDC). **Results**: Most FOS combinations exhibited additive or synergistic effects against clinical isolates. Synergy rates reached 72.7% for the FOS+CAZ-AVI combination (*K. pneumoniae*) and 65.0% for the FOS+COL combination (*P. aeruginosa*). An asymmetric synergistic interaction was identified for FOS+CAZ-AVI, with FOS enhancing the activity of CAZ-AVI more markedly than vice versa, especially in *K. pneumoniae*. Time-kill assays on selected isolates confirmed synergistic and bactericidal activity of FOS+CAZ-AVI and FOS+COL, and showed that bacterial regrowth observed with FOS, CAZ-AVI, and COL alone was suppressed in combination therapy. **Conclusions**: FOS-based combinations, particularly with CAZ-AVI and COL, demonstrated potent synergistic activity against MBL-producing *K. pneumoniae* and *P. aeruginosa*, supporting their potential utility in rational combination therapies for infections due to MBL-producing bacteria.

## 1. Introduction

Antimicrobial resistance (AMR) has emerged as one of the most alarming global health threats, with carbapenem-resistant Gram-negative bacteria posing a particularly severe challenge [1]. According to the World Health Organization (WHO), in 2023, approximately one in six confirmed bacterial infections worldwide were caused by bacteria resistant to antibiotics [2]. The Global Burden of Diseases, Injuries, and Risk Factors Study (GBD) estimated that 4.71 million deaths were associated with bacterial AMR in 2021, forecasting an estimated 8.22 million associated deaths globally in 2050 [1]. Among Gram-negative bacteria, resistance to carbapenems increased the most between 1990 and 2021, with associated deaths raising from 619,000 in 1990 to 1.03 million in 2021 [1].

Among the mechanisms driving carbapenem resistance, metallo-β-lactamases (MBLs) are of particular concern. These enzymes, of which New Delhi metallo-β-lactamase (NDM), Verona integron-encoded metallo-β-lactamase (VIM), and imipenemase (IMP) are most commonly found in clinical isolates, hydrolyze a broad spectrum of β-lactam antibiotics, including carbapenems, and are not inhibited by conventional β-lactamase inhibitors [3,4]. MBL-producing strains are frequently multidrug-resistant (MDR), and apart from the enzymatic inactivation of β-lactam antibiotics, often exhibit additional resistance strategies, including the overexpression of efflux pumps that actively expel antibiotics from the bacterial cell, alterations in membrane permeability that reduce drug uptake, and mutations in antibiotic target sites that diminish binding affinity [5,6,7,8,9]. These multifactorial resistance mechanisms contribute to the persistence and dissemination of highly resistant pathogens, leaving clinicians with few therapeutic options [3,4,7,8,9,10,11,12,13,14,15].

MBL-producing strains are widespread geographically and their prevalence is increasing globally and regionally. The NDM MBLs are the most frequent worldwide, with NDM-1 being the most common variant. The VIM MBLs are currently predominant in Europe, while IMP is predominant in Asia [3,4,10,11,16,17].

Two of the most clinically relevant MBL-producing pathogens are *Klebsiella pneumoniae* and *Pseudomonas aeruginosa*. These organisms are notorious for causing severe hospital-acquired infections and are increasingly resistant to multiple antibiotic classes [5,6,8,10]. Treatment options are limited and rely on last-resort agents such as colistin (COL), aminoglycosides (such as amikacin [AMI]), or newer drugs like aztreonam–avibactam (AZT-AVI), ceftazidime–avibactam (CAZ-AVI) plus aztreonam (AZT), and cefiderocol (FDC). However, these options may be limited by either toxicity, cost, and/or emergence of resistance [6,8,10,11,13].

In recent years, intravenous fosfomycin (FOS), which was discovered in the late 1960s [18], has re-emerged as a promising therapeutic agent, especially as combination partner to the antibiotic backbone for the treatment of various types of difficult-to-treat infections, often involving MDR bacteria [18,19,20,21,22,23,24]. FOS inhibits bacterial cell wall synthesis by targeting MurA, an enzyme involved in the earlier steps of peptidoglycan biosynthesis [18]. It offers several advantages: broad-spectrum activity against both Gram-positive and Gram-negative pathogens (including MBL producers), good uptake by bacterial cells, excellent tissue and body distribution, antibiofilm and intracellular activity, and a favorable safety profile [25,26,27,28,29,30,31,32]. Most notably, FOS exhibits broad synergistic activity with most antibiotic classes, particularly β-lactams [33]. In this context, several in vitro and animal model studies indicate that combinations involving FOS may enhance antimicrobial efficacy and suppress resistance development compared to monotherapy [10,19,21,22,26,34,35]. Although some in vitro synergy evaluations of FOS combinations against Gram-negative MBL producers have been reported [26,29,36,37,38,39,40,41,42,43] and recent clinical studies indicate clinical effectiveness of regimens including FOS in the treatment of MBL-producing Enterobacterales [44,45,46,47], data—especially from randomized clinical trials—are still limited [13,48,49].

This study aimed to evaluate the in vitro activity of FOS in combination with older and newer antimicrobials against MBL-producing *K. pneumoniae* (*n* = 22) and *P. aeruginosa* (*n* = 20) clinical isolates. These findings intend to inform rational combination therapy strategies including FOS for treating infections caused by MBL-producing Gram-negative bacteria.

## 2. Results

### 2.1. Bacterial Isolates’ Characteristics

A total of 42 MBL-producing clinical isolates were evaluated, including 22 *K. pneumoniae* and 20 *P. aeruginosa* isolates. Individual bacterial isolates are described in Table S1 (*K. pneumoniae*) and Table S2 (*P. aeruginosa*). Of the *K. pneumoniae* isolates, most (16/22 [72.7%]) carried an NDM-encoding gene (Table S1). In contrast, most (13/20 [65.0%]) *P. aeruginosa* clinical isolates carried a VIM-encoding gene (Table S2). Eight (36.4%) of *K. pneumoniae* isolates harbored more than one β-lactamase gene (two to four genes, including NDM, VIM, extended-spectrum beta-lactamase [ESBL], Temoneira β-lactamase [TEM], cefotaximase-Munich [CTX-M], and/or oxacillinase-48 [OXA-48]; Table S1), while only one (5.0%) of *P. aeruginosa* isolates expressed another β-lactamase gene (IMP-2 + OXA-35; Table S2). The complete antimicrobial susceptibility and genotypic profiles of individual isolates are provided in Table S1 (*K. pneumoniae*) and Table S2 (*P. aeruginosa*). Of the 42 investigated clinical isolates, 24 (57.1%) were MDR, defined as non-susceptibility to at least one agent in three or more antimicrobial categories [50], including 16/22 (72.7%) *K. pneumoniae* (Table S1) and 8/20 (40.0%) *P. aeruginosa* (Table S2) MDR isolates.

The minimum inhibitory concentration (MIC) of the tested antibiotics (FOS, MER, COL, AMI, AZT, CAZ-AVI, FDC and AZT-AVI) against these clinical isolates were determined by respective reference methods (see Section 4.2). MIC characteristics (MIC_50_, MIC_90_, MIC range) of the respective antibiotics tested alone, and susceptibility and resistance rates according to EUCAST (bracketed) breakpoints in the tested populations of *K. pneumoniae* and *P. aeruginosa* isolates are summarized in Table 1.

Across all 42 pathogens and antimicrobial drugs tested, MICs ranged from 0.06 to 1024 mg/L, and MIC_50_ and MIC_90_ from 0.5 to 1024 mg/L (Table 1). Most isolates (81.8% *K. pneumoniae* and 90.0% *P. aeruginosa* isolates) were classified as FOS wild type (labeled ‘S’ in Table 1) based on the species-specific epidemiological cut-off (ECOFF) value. Susceptibility to the other antibiotics ranged from 9.1% to 100% across the tested clinical isolates (Table 1). As expected, MER and CAZ-AVI showed high rates of resistance against both species (≥85.0%). Notably, 45.5% of MBL-producing *K. pneumoniae* were resistant to FDC, while all 22 *K. pneumoniae* isolates were susceptible to AZT-AVI.

### 2.2. Synergistic Activity of FOS Combinations Using Agar Dilution Checkerboard Assays

To evaluate the potential synergistic antimicrobial activity of the combination of FOS with other antimicrobial drugs, agar dilution-based checkerboard assays were performed as previously described [38]. Interactions between the combined antimicrobial agents were assessed based on the calculated fractional inhibitory concentration index (FICI).

Except for the FOS+COL combination in *K. pneumoniae*, which showed mainly (86.4%) indifference, all FOS combinations showed a positive interaction (additivity or synergy) in their antimicrobial activity against at least 50% of the tested *K. pneumoniae* and *P. aeruginosa* isolates (Table 2). Antagonism of FOS combinations was detected in only 2/42 (4.8%) isolates (one *K. pneumoniae* isolate with FOS+CAZ-AVI and one *P. aeruginosa* isolate with FOS+FDC; Table 2).

The most synergistic antimicrobial interactions were observed for FOS+CAZ-AVI (16/22 [72.7%] *K. pneumoniae* and 8/20 [40.0%] *P. aeruginosa* isolates), FOS+COL against *P. aeruginosa* (13/20 [65.0%] isolates), and FOS+AZT-AVI against *P. aeruginosa* (11/20 [55.0%] isolates), followed by FOS+MER (7/22 [31.8%] *K. pneumoniae* and 6/20 [30.0%] *P. aeruginosa* isolates), and FOS+FDC and FOS+AZT-AVI against *K. pneumoniae* (7/22 [31.8%] isolates each).

The fold reduction in FOS MICs used in combination vs. alone reached up to 32- to 2133-fold for *K. pneumoniae* and 2- to 512-fold for *P. aeruginosa*. In comparison, the MICs of antibiotic partners used in combination with FOS vs. when used alone reached up to 16- to 1024-fold for *K. pneumoniae* and 4- to 1024-fold for *P. aeruginosa* (Table 2).

Individual susceptible breakpoint index (SBPI) values for all antimicrobial combinations are shown in Table S3 (*K. pneumoniae*) and Table S4 (*P. aeruginosa*). Mean and median SBPI were >2 for all combinations, indicating that combinations were overall better than each agent alone. For each antimicrobial combination, SBPI varied among the individual bacterial isolates, ranging from 0.14 to 8194 across all antimicrobial combinations and tested isolates (Tables S3 and S4). The SBPI variability for an antimicrobial combination was sometimes striking. For instance, while 19/22 (86.4%) *K. pneumoniae* isolates were indifferent (according to calculated FICI; Table 2) to the FOS+COL combination (with SBPI ranging from 2.13 to 16.3; Table S3), 3/22 (13.6%) *K. pneumoniae* isolates were highly susceptible to this antimicrobial combination, with SBPI of 8194 (Table S3).

A closer evaluation of the MIC fold-reduction rates of FOS combinations (Table 2) revealed resensitization of isolates initially tested resistant to the respective single antibiotics. The most pronounced effects were observed for MER (46.3% and 30.0% susceptibility restoration rates in *K. pneumoniae* and *P. aeruginosa*, respectively), CAZ-AVI (90.9% and 75.0% susceptibility restoration rates in *K. pneumoniae* and *P. aeruginosa*, respectively), AZT (30.0% susceptibility restoration rate in *P. aeruginosa*), and FDC (31.9% susceptibility restoration rate in *K. pneumoniae*).

### 2.3. Directionality of FOS and CAZ-AVI Antibiotic Interaction

**Figure 1:**
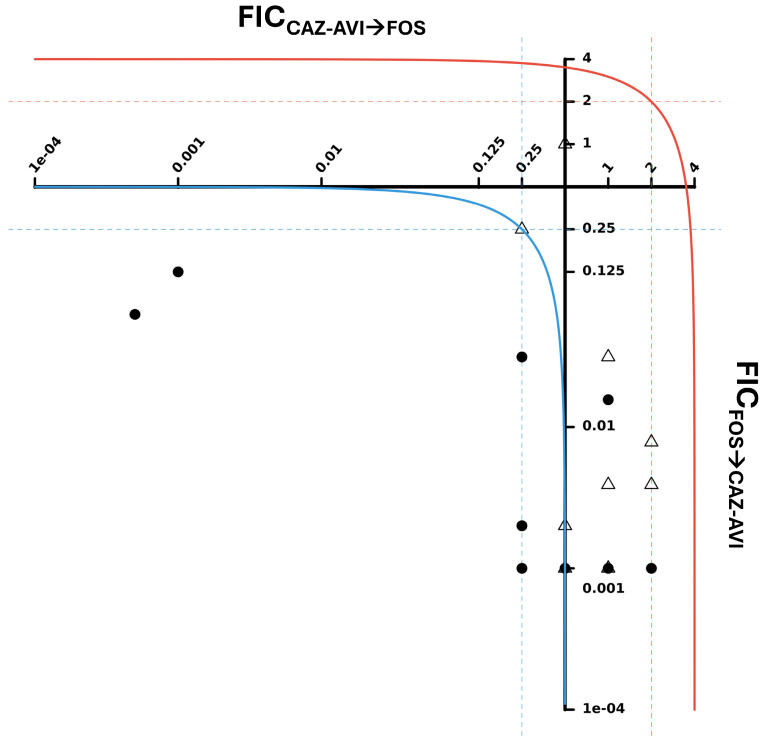
Bidirectional representation of individual fractional inhibitory concentration (FIC) values of FOS and CAZ-AVI antibiotics interactions. FIC_CAZ-AVI→FOS_ on the *x*-axis represents the directional effect of CAZ-AVI on FOS, while FIC_FOS→CAZ-AVI_ on the *y*-axis represents the directional effect of FOS on CAZ-AVI against *K. pneumoniae* (closed circles) and *P. aeruginosa* (open triangles) clinical isolates. Values are represented on log_2_ axes starting at 0.5, so that values to the left represent positive effects (synergy) vs. negative effects to the right (antagonism). The dotted lines indicate the limits of synergy (0.25) and antagonism (2.0) in the individual FIC representation (*blue* for synergy, *red* for antagonism). The continuous lines represent the global fractional inhibitory concentration index (FICI) limits of synergy (*blue*) and antagonism (*red*) (FICI = 0.5 for synergy; FICI ≥ 4 for antagonism). Individual FIC values for *K. pneumoniae* and *P. aeruginosa* clinical isolates are shown in Table S5 and Table S6, respectively. It should be noted that the FIC_CAZ-AVI→FOS_ value of the *K. pneumoniae* isolate 19-17-11 is not represented in Figure 1, as it lies outside the displayed range limits of the graph (FIC_CAZ-AVI→FOS_ = 32.0; see Table S5). Abbreviations: CAZ-AVI, ceftazidime–avibactam; FIC, fractional inhibitory concentration; FICI, fractional inhibitory concentration index; FOS, fosfomycin.

We further evaluated the synergistic activity of the antibiotic combination showing the strongest effect against both *K. pneumoniae* and *P. aeruginosa* (i.e., FOS+CAZ-AVI, with antimicrobial synergy in 72.7% and 40.0% *K. pneumoniae* and *P. aeruginosa* isolates, respectively; Table 2). In particular, the directionality in the synergistic effect of FOS and CAZ-AVI antimicrobial interaction was investigated using a two-axis representation of the individual fractional inhibitory concentration (FIC) values, where FIC_CAZ-AVI→FOS_ is on the *x*-axis and FIC_FOS→CAZ-AVI_ on the *y*-axis (Figure 1), as described in the Materials and Methods Section 4.3.3. In this bidirectional representation of FIC values, unilateral synergy is indicated by FIC ≤ 0.25. This representation revealed a strong synergistic effect of FOS on the action of CAZ-AVI against both species (Figure 1, FIC_FOS→CAZ-AVI_ axis, closed circles and open triangles) and a weaker synergistic impact of CAZ-AVI on the action of FOS, mostly on *K. pneumoniae* isolates (Figure 1, FIC_CAZ-AVI→FOS_ axis, closed circles).

Individual FIC values are shown in Table S5 (*K. pneumoniae*) and Table S6 (*P. aeruginosa*), highlighting directional synergistic effects (FIC ≤ 0.25). An asymmetric synergistic effect was evidenced, with CAZ-AVI benefiting more from the synergy promoted by FOS in 22/22 (100%) *K. pneumoniae* and 18/20 (90.0%) *P. aeruginosa* isolates (Tables S5 and S6, column FIC_FOS→CAZ-AVI_). In comparison, a directional synergistic effect of CAZ-AVI on FOS antimicrobial activity was observed in only 8/22 (36.4%) *K. pneumoniae* and 1/20 (5.0%) *P. aeruginosa* isolates (Tables S5 and S6, column FIC_CAZ-AVI→FOS_).

This directional synergistic effect of FOS over CAZ-AVI was also evidenced by the dramatic reduction in CAZ-AVI MIC by more than 1024-fold in most (16/22 [72.7%] *K. pneumoniae* and 11/20 [55.0%] *P. aeruginosa*) tested isolates. In contrast, FOS MIC values were reduced to a lesser extent (2–4-fold in 15/22 [68.2%] *K. pneumoniae* and 0–2-fold in 8/20 [40.0%] *P. aeruginosa* isolates).

### 2.4. Time-Kill Assays on Selected Isolates and the Most Synergistic Antimicrobial Combinations

To further evaluate synergistic FOS combinations and assess the rate and extent of bacterial killing over time, time-kill experiments were conducted on one isolate each of *K. pneumoniae* (NDM-producing clinical isolate 24-1-18; Table S1) and *P. aeruginosa* (VIM-producing clinical isolate 24-1-44; Table S2). These clinical isolates were randomly selected among those showing a clear synergistic response in the agar dilution checkerboard assay. Time-kill experiments were conducted with the antimicrobial agents presenting the strongest synergistic effect in combination (i.e., FOS+CAZ-AVI against *K. pneumoniae* and FOS+COL against *P. aeruginosa*; see Table 2), using free-fraction steady-state concentrations, as described in the Materials and Methods Section 4.3.5.

**Figure 2:**
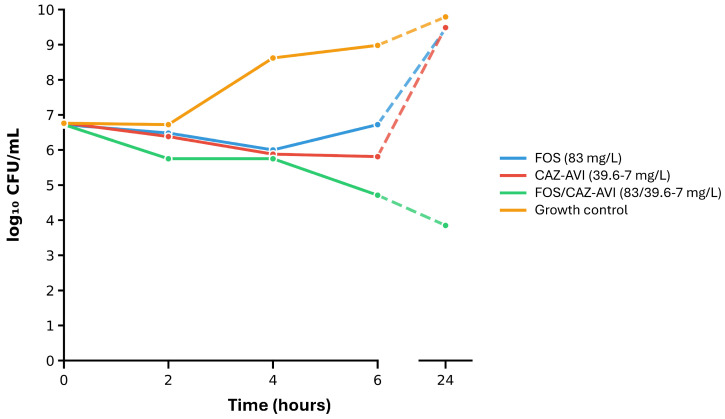
Time-kill assays on *K. pneumoniae* clinical isolate 24-1-18 (FOS MIC: 64 mg/L, CAZ-AVI MIC: 1024 mg/L). The following human free-fraction steady-state concentrations were used: 83 mg/L FOS [37], and 39.6 mg/L ceftazidime and 7 mg/L avibactam for CAZ-AVI [52]. A decrease in CFU/mL ≥2 log_10_ between the antimicrobial combination and its most active component was observed at 24 h for the FOS+CAZ-AVI condition (defining synergy). The decrease in CFU/mL at 24 h compared to the starting inoculum for FOS+CAZ-AVI was slightly <3 log_10_, thus not bactericidal. Time-kill assays were performed at least twice. Results of a representative experiment are displayed. Abbreviations: CAZ-AVI, ceftazidime–avibactam; FOS, fosfomycin.

**Figure 3:**
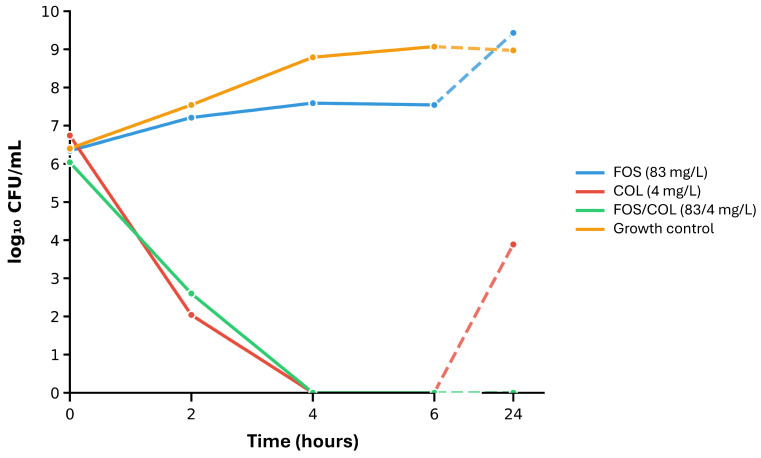
Time-kill assays on *P. aeruginosa* clinical isolate 24-1-44 (FOS MIC: 64 mg/L, COL MIC: 1 mg/L). The following human free-fraction steady-state concentrations were used: 83 mg/L FOS and 4.0 mg/L COL [37]. The effect of the FOS+COL combination was both synergistic and bactericidal at 24 h (synergy being defined as a decrease in CFU/mL ≥ 2 log_10_ between the antimicrobial combination and its most active component after 24 h, and a bactericidal effect being defined as a decrease in CFU/mL ≥ 3 log_10_ after 24 h compared to the starting inoculum). Time-kill assays were performed at least twice. Results of a representative experiment are displayed. Abbreviations: COL, colistin; FOS, fosfomycin.

Compared to the drug-free growth control, FOS alone exerted a weak or no antimicrobial effect on the growth of both *K. pneumoniae* and *P. aeruginosa* isolates (Figure 2 and Figure 3, blue curve vs. orange curve). Both isolates also showed regrowth in the presence of FOS alone after 24 h of incubation (Figure 2 and Figure 3, blue curve). CAZ-AVI alone showed a comparable effect on the growth of the *K. pneumoniae* isolate (Figure 2, red curve). In contrast, COL alone showed a strong antimicrobial effect on the growth of the *P. aeruginosa* isolate in the first hours of incubation. This initial growth inhibitory effect was however followed by a strong regrowth at 24 h (Figure 3, red curve), likely indicative of acquired resistance or heteroresistance.

In both FOS+CAZ-AVI and FOS+COL time-kill assays, no regrowth was observed after 24 h of incubation (Figure 2 and Figure 3, green curve). Both antimicrobial combinations were synergistic (defined as a ≥2 log_10_ decrease in CFU/mL between the antimicrobial combination and its most active component after 24 h). In the case of FOS+COL (Figure 3), the effect on the tested *P. aeruginosa* isolate was also bactericidal (defined as a ≥3 log_10_ decrease in CFU/mL after 24 h compared to the starting inoculum), while not quite reaching the bactericidal threshold for the FOS+CAZ-AVI combination against the *K. pneumoniae* isolate (2.89 log_10_ decrease in CFU/mL after 24 h compared to the starting inoculum).

## 3. Discussion

MBL-producing Gram-negative bacteria pose an increasing risk for public health, with only few therapeutic options available. FOS, which exhibits in vitro activity against MBL producers [25,26,27,28,29,30,31,32,53], has experienced a renaissance in recent years. It is primarily used as adjunctive to other antibiotics, thanks to its broad synergistic interactions, against difficult-to-treat infections caused by both MDR Gram-negative and Gram-positive pathogens [11,19,20,33]. The present study reports the results of an extensive analysis of the potential synergistic effect of FOS combined with other antimicrobial agents against 42 MBL-producing *K. pneumoniae* and *P. aeruginosa* clinical isolates collected at various clinical centers across Europe.

Among the tested isolates, the susceptibility to the old drugs FOS and COL was relatively high in both species (between 72.7% and 95.0%), as previously reported [54]. In contrast, susceptibility to MER was notably low for both *K. pneumoniae* and *P. aeruginosa* MBL isolates (13.6% and 15.0%, respectively), in agreement with previous studies [55,56]. Likewise, only 9.1% of the tested *K. pneumoniae* isolates were susceptible to CAZ-AVI, while AZT-AVI demonstrated 100% susceptibility against *K. pneumoniae*. Markedly, resistance to FDC among *K. pneumoniae* clinical strains was relatively high (45.5%). In this context, an even higher resistance rate (69.7%) was reported for NDM-producing Enterobacterales in the study by Baltas et al. [54], which may limit the use of FDC as monotherapy or empirically. In contrast, the in vitro activity of FDC against tested *P. aeruginosa* isolates was high with 80% susceptibility.

Agar dilution checkerboard experiments showed that, overall, most evaluated antimicrobial combinations exerted a positive (additive or synergistic) effect against clinical isolates. Most notably, synergy rates reached 72.7% for the FOS+CAZ-AVI combination against the 22 *K. pneumoniae* isolates and 65.0% for the FOS+COL combination against the 20 *P. aeruginosa* isolates.

Several previously published studies also demonstrated a synergy of the FOS+CAZ-AVI combination against MBL-producing *K. pneumoniae* isolates (NDM- and IMP-producers), with synergy rates ranging from 50.0% to 69.2% compared to CAZ-AVI alone [33,40,57,58], thus close to the 72.7% synergy observed for MBL-producing *K. pneumoniae* in the current report. These observations suggest that the FOS+CAZ-AVI combination may be a treatment option against MBL-producing *K. pneumoniae*. Importantly, to the best of our knowledge, the present study is the first to describe a synergistic effect of FOS+CAZ-AVI (40.0%) and FOS+AZT-AVI (55.0%) against MBL-producing *P. aeruginosa*. Both CAZ-AVI and AZT-AVI on their own exhibit only limited activity against MBL *P. aeruginosa*, as is suggested by the current findings (Table 1 and Table S2) and others’ [59,60]. The presented in vitro synergy data demonstrated overall positive interactions for both β-lactam/β-lactamase inhibitors (BLBLIs) in combination with FOS, resulting in a strong reduction in CAZ-AVI and AZT-AVI MICs. Accordingly, the combination of FOS+CAZ-AVI or FOS+AZT-AVI may be a promising option in the treatment of MBL-producing *P. aeruginosa*, especially in regions lacking access to FDC, which is the main treatment option for these infections. However, further in vitro experiments and, more importantly, clinical data are needed to confirm these findings and establish the therapeutic value of these FOS-containing combinations in clinical practice.

Data for synergistic effects of the FOS+COL combination against MBL-producing *P. aeruginosa* are also scarce. Two studies showed a synergy of the FOS+COL combination in carbapenem-resistant and MDR *P. aeruginosa* isolates, with synergy rates between 13.3% and 21.8% compared to COL alone [61,62]. These synergy rates are lower than that observed in MBL-producing *P. aeruginosa* in the present study (65.0%). However, both studies used different methods to test for synergistic effects and, furthermore, did not indicate whether the carbapenem-resistant and MDR *P. aeruginosa* included in the respective studies were MBL-producers [33,61,62]. These aspects might explain the apparent discrepancy with our data. Indeed, existing studies show a trend for a lower synergy rate of FOS combinations against non-MBL-producing carbapenem-resistant bacterial isolates. In one study, the FOS+CAZ-AVI-mediated synergy rate was 27.0% against carbapenemase-non-producing *K. pneumoniae* and 43.4% against class A serine-β-lactamase KPC-producing *K. pneumoniae* compared to 69.2% against class B MBL-producing *K. pneumoniae* (with 64.3% for NDM producers and 75.0% for IMP producers) [58]. In another study, the FOS+CAZ-AVI-mediated synergy rate for KPC-producing *K. pneumoniae* was 37.5% vs. 50.0% for NDM-producing *K. pneumoniae* [40].

Besides the promising results with BLBLIs, the FOS+MER combination also showed positive interactions in a considerable percentage of isolates from both species (60.0% to 63.6% positive interactions, considering synergy and additivity). In this context, a recent study using an in vitro pharmacokinetic model involving *K. pneumoniae* isolates with various resistance mechanisms (including VIM and NDM) against β-lactam antibiotics showed that the addition of MER to FOS dramatically reduced both the FOS *f*AUC/MIC (free area under the curve/MIC, i.e., the total drug exposure over time relative to the MIC) and *f*T > MIC (free time above MIC, i.e., the duration of drug concentration remaining > MIC) exposures required for bacteriostatic and bactericidal effects [35].

In line with other studies [57,63,64], the present results revealed a strong variability in the susceptibility to antimicrobial combinations across tested bacterial isolates (i.e., indifference, additivity, synergy, or—more rarely in the present study—antagonism), even among those with similar β-lactamase genotypes. This variability is likely due to the multifactorial features of resistance mechanisms acquired by each isolate [5,6,7,8,9], which are also targeted by combination therapy [65].

Given the interesting finding that the combination of FOS+CAZ-AVI produced a stronger reduction in CAZ-AVI MIC than in FOS MIC for both species, we investigated a potential directionality of the interaction of FOS and CAZ-AVI in all 42 MBL-producing bacterial isolates employing a two-axis plotting of individual FIC_CAZ-AVI→FOS_ and FIC_FOS→CAZ-AVI_ values, as previously described [66]. This analysis revealed an asymmetric interaction of antimicrobial partners, with CAZ-AVI benefitting most from the synergistic effect exerted by FOS (with 40/42 [95.2%] clinical isolates showing a synergistic effect of FOS on CAZ-AVI vs. only 9/42 [21.4%] with a synergistic effect of CAZ-AVI on FOS), especially against *K. pneumoniae* isolates. These data confirm an added value of interpreting individual FICs over FICIs to evaluate the directionality of antimicrobial effects [66], which is a common phenomenon in combination therapy [65,67,68,69]. For instance, in a recent pharmacokinetic/pharmacodynamic study of the FOS+MER combination, MER enhanced the potency of FOS against MDR and carbapenemase-producing *K. pneumoniae* [69].

In accordance with the results from the checkerboard experiments, time-kill assays on selected isolates and antimicrobial combinations (FOS+CAZ-AVI against an NDM-producing *K. pneumoniae* isolate, FOS+COL against a VIM-producing *P. aeruginosa* isolate) demonstrated synergistic (both FOS+CAZ-AVI and FOS+COL) and bactericidal (FOS+COL) effects. In both cases, the antimicrobial combination prevented bacterial regrowth and suppressed acquired resistance. These observations are in line with previous studies demonstrating the benefit of FOS combinations in increasing bactericidal activity and preventing the emergence of resistance [33,62,70].

The predominantly positive interactions between FOS and other antibiotics tested in this study may be explained by complementary mechanisms of action. For example, FOS+COL, which showed strong synergy against MBL *P. aeruginosa* in the present report, combines two antibiotic agents with distinct targets of the bacterial cell wall. FOS is a phosphonic acid derivative. It enters bacterial cells via specific transporters. Once in the cell, FOS inhibits MurA, an enzyme involved in the first committed step of peptidoglycan biosynthesis, which is essential for bacterial cell wall formation. By weakening the bacterial cell wall and contributing to bacterial cell lysis, FOS is an acknowledge bactericidal agent [18,65]. On the other hand, COL is a polymyxin antibiotic targeting lipopolysaccharides (LPS) in the outer membrane of Gram-negative bacteria. By doing so, it disrupts the outer cell membrane integrity, thereby increasing cell permeability [65]. Thus, it may be assumed that the FOS+COL synergy against the MBL-producing *P. aeruginosa* isolates observed in the present study arose from enhanced intracellular uptake of FOS (independently of FOS transporters) through COL-mediated disruption of the outer membrane, and dual cell wall targeting (via COL binding to LPS and FOS-induced inhibition of peptidoglycan synthesis). This dual cell wall targeting mechanism may result in an enhanced bactericidal activity, even in MBL-producing strains, given the non-β-lactam mechanisms of both FOS and COL. A similar dual cell wall targeting mechanism might explain the synergy observed with FOS+CAZ-AVI (inhibition of early cell wall synthesis by FOS and inhibition of late-stage peptidoglycan cross-linking by ceftazidime) [65,71], with a predominant effect of FOS on enhancing the action of CAZ-AVI, based on the directionality of the FOS+CAZ-AVI interaction observed in the present study.

Taken together, the presented results indicate that FOS may be a promising partner in antimicrobial combinations against MBL-producing Gram-negative bacteria, particularly in combination with novel β-lactam antibiotics. Further studies are needed to confirm existing in vitro data and, importantly, expand the so far limited clinical data on FOS as a potential option in the treatment of infections caused by MBL-producing Enterobacterales (such as *K. pneumoniae*) and *P. aeruginosa* [29,33,36,37,38,39,40,43,44,45,46,47,58]. Future studies should also address the antibiofilm capacity of FOS combinations against these MBL producers.

This study presents several notable strengths, including a comprehensive investigation of the synergistic activity of FOS combinations using diverse assays and methods (agar dilution method, SBPI, assessment of the directionality of the antimicrobial effect of each combination partner based on individual FICs, time-kill assays), and the multicenter nature of the study, which assessed isolates from several countries across Europe.

This study has several limitations. First, the total number of MBL-producing bacterial isolates included in the study was relatively small (*n* = 42). Second, MBL isolates were selected by each center, and thus are not epidemiologically representative. Third, time-kill assays were conducted on only one isolate each of *K. pneumoniae* and *P. aeruginosa*. Time-kill assays on more isolates should be performed to better evaluate the rate and extent of bacterial killing over time by FOS combinations. Fourth, this study focused on MBL-producing *K. pneumoniae* and *P. aeruginosa* strains. Accordingly, the synergy data of FOS combinations presented in this study are only representative of this class of pathogens. Finally, one should keep in mind that in vitro antibiotic combination exposure data may not directly translate into clinical outcomes. Therefore, clinical validation of these in vitro results is warranted. Nonetheless, the current findings provide a strong mechanistic rationale and important implications for therapeutic decision-making.

## 4. Materials and Methods

### 4.1. Bacterial Isolates

A total of 42 MBL-producing clinical isolates, including 22 *K. pneumoniae* and 20 *P. aeruginosa*, were obtained from routine clinical examination of patients at five centers across Europe. The 42 strains were isolated between 2001 and 2024 and selected for this study by the following participating centers: Paul-Ehrlich-Society for Infection Therapy (Cologne, Germany; *n* = 6 isolates collected between 2019 and 2020), Raymond-Poincaré University Hospital (Garches, France; *n* = 9 isolates collected between 2021 and 2024), National and Kapodistrian University of Athens (Athens, Greece; *n* = 9 isolates collected between 2015 and 2024), National Medicines Institute (Warsaw, Poland; *n* = 12 isolates collected between 2001 and 2015), and Cotugno Hospital, Azienda Ospedaliera dei Colli (Naples, Italy; *n* = 6 isolates collected between 2023 and 2024). The present study was conducted between June 2024 and October 2025.

Isolates were identified either by biochemical profiling using the VITEK^®^ 2 automated identification system (bioMérieux, Marcy l’Étoile, France) or by matrix-assisted laser desorption/ionization time-of-flight method (MALDI-TOF) employing VITEK^®^ MS (bioMérieux, Nürtingen, Germany), Microflex LT (Bruker Daltonics, Bremen, Germany) or MALDI Biotyper^®^ (Bruker Daltonics, Billerica, MA, USA).

Screening for class A and B carbapenemases was conducted using the modified Hodge test (MHT) to detect carbapenemase activity due to its excellent sensitivity for detecting enterobacterial isolates producing KPC [72]. The combined phenylboronic acid (PBA) and ethylenediaminetetraacetic acid (EDTA) double-disk test, with meropenem (±ceftazidime) as the substrate, was also performed [73,74]. Other methods for confirming carbapenemase production were employed, including carbapenem hydrolysis tests, immunoenzymatic assays, or molecular detection assays (Xpert^®^ Carba-R, Cepheid, Maurens-Scopont, France). ESBL co-production was assessed using a modified Clinical and Laboratory Standards Institute (CLSI) ESBL combined disk test [75,76] or using the double-disk diffusion test with ceftazidime (30 µg), cefotaxime (30 µg), and amoxicillin-clavulanic acid (20/10 µg) disks [77]. Total DNA from the clinical isolates was extracted by the boiling method [78]. Polymerase chain reaction (PCR) with specific primers was used to detect genes encoding carbapenemases (*bla*_KPC_, *bla*_NDM_, *bla*_VIM_) [79,80,81,82], as well as ESBLs (*bla*_SHV_, *bla*_TEM_, *bla*_CTX-M_ group 1) [83], as previously described [84]. Additionally, whole genome sequencing (WGS) using Illumina HiSeq (Illumina, San Diego, CA, USA) was performed to detect specific variants of the carbapenemase genes.

### 4.2. Antimicrobial Susceptibility Testing

Susceptibility testing was performed for each antimicrobial drug using reference methods.

Broth microdilution with in-house prepared plates (ISO 20776-1:2019; [85]) was used for susceptibility testing of the following antibiotics: amikacin (AMI) (0.12–128 mg/L), aztreonam (AZT) (0.06–64 mg/L), aztreonam–avibactam (AZT-AVI) (0.06/4–32/4 mg/L), colistin (COL) (0.03–32 mg/L), meropenem (MER) (0.03–32 mg/L) and ceftazidime–avibactam (CAZ-AVI) (1/4–512/4 mg/L). The concentration of the β-lactamase-inhibitor avibactam was fixed at 4 mg/L. The fixed concentration was not separately displayed in the minimal inhibitory concentration (MIC) results.

Fosfomycin (FOS) susceptibility was investigated by agar dilution (0.25–512 mg/L) in the presence of 25 mg/L glucose-6-phosphate (CLSI M07-A10, 2015; [86]).

For susceptibility testing of cefiderocol (FDC), disk diffusion is recommended by the European Committee of Antimicrobial Susceptibility Testing (EUCAST) [87]. However, this method only classifies isolates into susceptible and resistant but fails to indicate a MIC, which is needed for the calculation of the fractional inhibitory concentration index (FICI). In addition to disk diffusion, the commercially available UMIC cefiderocol test kit (Bruker, Bornheim, Germany) was used with 0.03–32 mg/L FDC and iron-depleted cation-adjusted Mueller–Hinton broth (Bruker, Bornheim, Germany). Both methods were compared with regard to the classification of all isolates into susceptible or resistant.

All experiments were performed at least twice. Reference strains *Escherichia coli* (*E. coli*) ATCC 25922 and *P. aeruginosa* ATCC 27853 were used as quality controls.

### 4.3. Evaluation of Combined Antimicrobial Activity

#### 4.3.1. Agar Dilution Checkerboard Testing

Agar dilution-based checkerboard was performed according to Erturk Sengel et al. [38]. FOS was combined with the different antimicrobial drugs stated above, in plates including cation-adjusted Mueller–Hinton agar (Merck, Darmstadt, Germany) supplemented with 25 mg/L glucose-6-phosphate (Merck, Darmstadt, Germany). Two-fold dilutions of drug concentrations starting from four-fold above their MIC values were tested as follows, based on the MIC results of the single substances against *K. pneumoniae* and *P. aeruginosa*. AMI and AZT with a range of 0.12–64 mg/L each were tested in combination with a FOS range of 0.25–512 mg/L against *K. pneumoniae* (AMI/FOS only) and *P. aeruginosa* (AZT/FOS only) isolates. MER/FOS was tested for both species using the same concentration ranges as for AMI/FOS or AZT/FOS (0.12/0.25–64/512). The combination of FDC and FOS required a different range for both substances (0.03/0.12–4/256 mg/L for FDC/FOS) for *K. pneumoniae* and *P. aeruginosa*. Similar to broth microdilution, the concentration of avibactam in each combination was fixed at 4 mg/L and was not separately displayed in the MIC results. Concentration ranges of the avibactam combinations were as follows: AZT-AVI/FOS, 0.015/4/0.015–64/4/512 mg/L (both species); CAZ-AVI/FOS, 0.015/4/0.015–32/4/512 mg/L (*K. pneumoniae*) and 0.12/4/0.25–64/4/512 mg/L (*P. aeruginosa*). The ranges for COL and FOS combinations were also adjusted for each species: COL/FOS of 0.015/0.015–8/32 mg/L for *K. pneumoniae*, and of 0.12/0.25–64/512 mg/L for *P. aeruginosa*. All experiments were performed at least twice. Reference strains *E. coli* ATCC 25922 and *P. aeruginosa* ATCC 27853 were used as quality controls.

#### 4.3.2. Fractional Inhibitory Concentration Index (FICI) Calculation

The global interaction between combined antimicrobial agents A and B was assessed by calculating the fractional inhibitory concentration index (FICI) based on the individual fractional inhibitory concentration (FIC) values obtained from the agar dilution checkerboard results, as follows:

FICI  =  FIC_A_ + FIC_B_

where

FIC_A_ = MIC A in combination/MIC A alone.

FIC_B_ = MIC B in combination/MIC B alone.

To classify the antimicrobial efficacy of the combination, FICIs were interpreted as follows: FICI ≤ 0.5, “synergistic”; FICI ≤ 1, “additive”; FICI ≤ 4, “no interaction/indifferent”; FICI > 4, “antagonistic” [88,89].

#### 4.3.3. Directionality of Antimicrobial Combination Effects

While assessing the global efficacy of antimicrobial combinations (synergism, additivity, indifference, or antagonism), FICIs do not provide information on the directionality of the interaction (i.e., which agent impacts the other one). This was evaluated by graphically representing the respective FIC data in a two-axis plot where FIC_B→A_ is on the *x*-axis and FIC_A→B_ on the *y*-axis (where A and B are the two tested compounds), as described by Fatsis-Kavalopoulos et al. [66]. Assuming a bilateral interaction (FIC_B→A_ = FIC_A→B_), a synergistic FICI < 0.5 translates to FIC_B→A_ = FIC_A→B_ = 0.25 and an antagonistic FICI > 4 translates to FIC_B→A_ = FIC_A→B_ = 2. Accordingly, the thresholds of unilateral interactions are interpreted as follows: FIC_A→B_ ≤ 0.25, “synergy” (compound A promotes the action of compound B); 0.25 < FIC_A→B_ < 2, “additivity/indifference” (compounds A and B are additive/indifferent); FIC_A→B_ = 0.5, threshold of “additivity/indifference”; FIC_A→B_ ≥ 2, “antagonism” (compound A inhibits the action of compound B) [66].

#### 4.3.4. Susceptibility Breakpoint Index (SBPI) Evaluation

The susceptible breakpoint index (SBPI) evaluates the MIC of antimicrobial agents tested in combination relative to their individual susceptibility breakpoints. The SBPI appears to be more discriminatory than the FICI [90], and thus may be more clinically relevant. The SBPIs were calculated using EUCAST clinical breakpoints or breakpoints in brackets (or for FOS, the species-specific epidemiological cut-off [ECOFF] values) (see Table 1 for details) [91], as follows: 

SBPI = (susceptible breakpoint A/combination MIC A)  +  (susceptible breakpoint B/combination MIC B)

An SBPI  ≥ 2 indicates that the MICs of combined antimicrobials A and B are equal or lower than their respective susceptibility breakpoints (or ECOFFs in case of FOS). Thus, the greater the SBPI value, the more effective the antimicrobial combination is [90,92].

#### 4.3.5. Time-Kill Assays

To further evaluate antimicrobial combination synergistic activity and assess the rate and extent of bacterial killing over time, time-kill assays were conducted on one randomly selected isolate each of *K. pneumoniae* and *P. aeruginosa*. Both selected isolates were tested with the respective most synergistic combination based on the agar dilution checkerboard experiments. Antibiotic concentrations used during time-kill assays were human mean steady-state concentrations of non-protein-bound (free) drug calculated from reported literature (based on the area-under-the-antibiotic concentration time-curve in serum or plasma over 24 h divided by 24 h [AUC_0–24_/24 h]). The following free-fraction steady-state concentrations were used: 83 mg/L FOS, 4.0 mg/L COL [37], and for CAZ-AVI, 39.6 mg/L ceftazidime and 7 mg/L avibactam [52]. Selected bacterial isolates were exposed to the test antibiotics in broth media. Samples were collected at 0, 2, 4, 6 and 24 h, serially diluted, and plated to determine viable counts (colony forming units [CFU]/mL). Data were expressed as log_10_ CFU/mL.

Synergy was defined as a ≥2 log_10_ decrease in CFU/mL between the antimicrobial combination and its most active component after 24 h. A bactericidal effect was defined as a ≥3 log_10_ decrease in CFU/mL after 24 h compared to the starting inoculum [37]. Time-kill assays were performed at least twice.

### 4.4. Data Analysis and Software

Continuous variables were analyzed using descriptive statistics (mean, standard deviation [SD], median, interquartile range [IQR], minimum and maximum). Figures were prepared using Python (version 3.10.18) [93] and R software (version 4.4.3) [94].

## 5. Conclusions

This study revealed potent synergistic activity of FOS combinations—especially with CAZ-AVI and COL—against MBL-producing *K. pneumoniae* and *P. aeruginosa*. These findings support a potential benefit of FOS in combination regimens for the treatment of infections caused by MBL-producing Gram-negative bacteria. Further in vitro investigations involving a larger, more diverse, and epidemiology-relevant set of MBL-producing pathogens and, particularly, clinical data are warranted to validate and expand these results.

## Figures and Tables

**Table 1:** Minimum inhibitory concentration (MIC) of the *K. pneumoniae* and *P. aeruginosa* clinical isolates used in this study, as determined by reference method (broth microdilution, unless indicated otherwise).

	MIC [mg/L]			EUCAST Breakpoints		
Antibiotic	MIC_50_	MIC_90_	MIC Range	MIC Breakpoint [mg/L]	S [%]	R [%]
***K. pneumoniae* (*n* = 22)**						
FOS ^a^	32	1024	1–1024	≤128 ^c^	81.8 *	18.2 *
AMI	16	256	1–256	≤(8) ^d^	31.8	68.2
MER	64	64	0.5–64	≤8 ^e^	13.6	86.4
COL	0.5	64	0.5–64	≤(2) ^d^	72.7	27.3
CAZ-AVI	1024	1024	1–1024	≤8	9.1	90.9
AZT-AVI	0.25	0.5	0.06–1	≤4	100	0
FDC ^b^	1	16	0.06–32	≤2	54.5	45.5
***P. aeruginosa* (*n* = 20)**						
FOS ^a^	16	128	4–1024	≤256 ^c^	90.0 **	10.0 **
MER	64	64	0.5–64	≤8 ^e^	15.0	85.0
COL	1	4	1–16	≤(4) ^d^	95.0	5.0
AZT	16	32	4–128	≤16 ^e^	60.0	40.0
CAZ-AVI	128	1024	1–1024	≤8	15.0	85.0
AZT-AVI	16	32	0.06–64	IE	-	-
FDC ^b^	0.5	4	0.06–4	≤2	80.0	20.0

^a^ MIC determined by agar dilution method. ^b^ MIC determined employing UMIC^®^ microdilution. ^c^ Epidemiological cut-off (species-specific). ^d^ Breakpoint in brackets. ^e^ Increased exposure (I) breakpoint. * Proportion of isolates belonging to the wild-type population (S) and non-wild-type population (R); note: based on the Comité de l’Antibiogramme de la Société Française de Microbiologie (CA-SFM) breakpoint, for Enterobacterales of 32 mg/L, 63.6% of isolates were susceptible to fosfomycin [51]. ** proportion of isolates belonging to the wild-type population (S) and non-wild-type population (R). Abbreviations: AMI, amikacin; AZT, aztreonam; AZT-AVI, aztreonam–avibactam; COL, colistin; CAZ-AVI, ceftazidime–avibactam; EUCAST, European Committee on Antimicrobial Susceptibility Testing; FDC, cefiderocol; FOS, fosfomycin; I, increased exposure (susceptible; sometimes referred to as ‘intermediate’); IE, insufficient evidence; MER, meropenem; MIC, minimal inhibitory concentration; MIC_50_, MIC at which 50% of a bacterial population is inhibited from growth; MIC_90_, MIC at which 90% of a bacterial population is inhibited from growth; R, resistant; S, susceptible.

**Table 2:** Agar dilution checkerboard results of FOS combinations for *K. pneumoniae* and *P. aeruginosa clinical* isolates.

Combination	FICI Category (*n* [%]) *				MIC Fold-Reduction (FOS/Partner)
	Synergy	Additivity	Indifference	Antagonism	
***K. pneumoniae* (*n* = 22)**					
FOS+MER ^a^	7 (31.8)	7 (31.8)	8 (36.4)	0 (0)	0–128/0–128
FOS+COL ^a^	3 (13.6)	0 (0)	19 (86.4)	0 (0)	0–512/0–16
FOS+AMI ^a^	3 (13.6)	8 (36.4)	11 (50.0)	0 (0)	0–32/0–32
FOS+CAZ-AVI ^a^	**16 (72.7)**	1 (4.5)	4 (18.2)	1 (4.5)	0–2048/8–1024
FOS+FDC ^a^	7 (31.8)	5 (22.7)	10 (45.5)	0 (0)	0–64/0–16
FOS+AZT-AVI ^a^	7 (31.8)	5 (22.7)	10 (45.5)	0 (0)	0–2133/0–16
***P. aeruginosa* (*n* = 20)**					
FOS+MER ^b^	6 (30.0)	6 (30.0)	8 (40.0)	0 (0)	0–8/0–16
FOS+COL ^b^	**13 (65.0)**	5 (25.0)	2 (10.0)	0 (0)	0–256/0–32
FOS+AZT ^b^	3 (15.0)	8 (40.0)	9 (45.0)	0 (0)	0–8/0–32
FOS+CAZ-AVI ^b^	8 (40.0)	7 (35.0)	5 (25.0)	0 (0)	0–2/0–1024
FOS+FDC ^b^	1 (5.0)	9 (45.0)	9 (45.0)	1 (5.0)	0–512/0–4
FOS+AZT-AVI ^b^	11 (55.0)	6 (30.0)	3 (15.0)	0 (0)	0–8/0–32

^a^ MIC of one or both partner antibiotics not within tested concentration range (MER: *n* = 4; COL: *n* = 10; AMI: *n* = 7; CAZ-AVI: *n* = 21; FDC: *n* = 5; AZT-AVI: *n* = 6). ^b^ MIC of one or both partner antibiotics not within tested concentration range (MER: *n* = 9; COL: *n* = 2; AZT: *n* = 3; CAZ-AVI: *n* = 15; FDC: *n* = 13; AZT-AVI: *n* = 4). * FICIs were interpreted as follows: FICI ≤ 0.5, “synergy”; FICI ≤ 1, “additivity”; FICI ≤ 4, “indifference”; FICI > 4, “antagonism”. The most synergistic combinations against *K. pneumoniae* (FOS+CAZ-AVI) and *P. aeruginosa* (FOS+COL) isolates are indicated in bold. Abbreviations: AMI, amikacin; AZT, aztreonam; AZT-AVI, aztreonam–avibactam; COL, colistin; CAZ-AVI, ceftazidime–avibactam; FDC, cefiderocol; FICI, fractional inhibitory concentration index; FOS, Fosfomycin; MER, meropenem; MIC, minimal inhibitory concentration.

## Data Availability

The original contributions presented in this study are included in the article and Supplementary Material. Further inquiries can be directed to the corresponding author.

## Supplementary Materials

The following supporting information can be downloaded at: https://www.mdpi.com/article/10.3390/antibiotics14121247/s1, Table S1: Minimum inhibitory concentration (MIC) determination of carbapenemase-producing *K. pneumoniae* clinical isolates by reference method; Table S2: Minimum inhibitory concentration (MIC) determination of carbapenemase-producing *P. aeruginosa* clinical isolates by reference method; Table S3: Susceptible breakpoint index (SBPI) of carbapenemase-producing *K. pneumoniae* clinical isolates from AD checkerboard experiments; Table S4: Susceptible breakpoint index (SBPI) of carbapenemase-producing *P. aeruginosa* clinical isolates from AD checkerboard experiments; Table S5: Individual fractional inhibitory concentration (FIC) values of the combination CAZ-AVI and fosfomycin against MBL-producing *K. pneumoniae* clinical isolates; Table S6: Individual fractional inhibitory concentration (FIC) values of the combination CAZ-AVI and fosfomycin against MBL-producing *P. aeruginosa* clinical isolates.

## Abbreviations

The following abbreviations are used in this manuscript:

AMI	Amikacin
AZT	Aztreonam
AZT-AVI	Aztreonam–avibactam
BLBLI	β-lactam/β-lactamase inhibitor
CLSI	Clinical and Laboratory Standards Institute
COL	Colistin
CTX-M	Cefotaximase Munich
CAZ-AVI	Ceftazidime–avibactam
ECOFF	Epidemiological cut-off
EDTA	Ethylenediaminetetraacetic acid
ESBL	Extended-spectrum-β-lactamase
EUCAST	European Committee on Antimicrobial Susceptibility Testing
FDC	Cefiderocol
FIC	Fractional inhibitory concentration
FICI	Fractional inhibitory concentration index
FOS	Fosfomycin
GIM	German imipenemase
IMP	Imipenemase
KPC	*Klebsiella pneumoniae* carbapenemase
MALDI-TOF	Matrix-assisted laser desorption/ionization–time-of-flight
MBL	Metallo-β-lactamase
MDR	Multidrug resistant
MER	Meropenem
MHT	Modified Hodge test
MIC	Minimal inhibitory concentration
NDM	New Delhi metallo-β-lactamase
OXA	Oxacillinase
PBA	Phenylboronic acid
PCR	Polymerase chain reaction
SBPI	Susceptible breakpoint index
TEM	Temoneira β-lactamase
VIM	Verona integron-encoded metallo-β-lactamase
WGS	Whole genome sequencing
