# Supplementary material for: In Vitro Evaluation of Fosfomycin Combinations Against Metallo-β-Lactamase-Producing Klebsiella pneumoniae and Pseudomonas aeruginosa Clinical Isolates
Source: Antibiotics (Basel). 2025 Dec 10;14(12):1247. doi: 10.3390/antibiotics14121247 (PMC12729803; doi:10.3390/antibiotics14121247)
Supplement: Supplementary file 1 [file antibiotics-14-01247-s001.zip › antibiotics-4023603-supplementary.pdf]

Supplementary tables

Table S1. Minimum inhibitory concentration (MIC) determination of carbapenemase-producing *K. pneumoniae* clinical isolates by reference method.

Strain	β-lactamase	MIC [mg/L] (category)						
		FOS [a]	AMI	MER	COL	CAZ-AVI	AZT-AVI	FDC [b]
24-1-1 *	VIM+ESBL	1024 (NWT)	16 (R)	64 (R)	32 (R)	1024 (R)	0.25 (S)	1 (S)
24-1-2 *	NDM+ESBL	32 (WT)	32 (R)	64 (R)	4 (R)	1024 (R)	0.25 (S)	4 (R)
24-1-3 *	VIM+TEM	1024 (NWT)	256 (R)	64 (R)	0.5 (S)	1024 (R)	0.5 (S)	32 (R)
24-1-6 *	NDM+VIM+CTX-M+TEM	64(WT)	16 (R)	64 (R)	32 (R)	1024 (R)	0.25 (S)	8 (R)
24-1-12 *	NDM+CTX-M+TEM	1024 (NWT)	256 (R)	64 (R)	64 (R)	1024 (R)	1 (S)	16 (R)
24-1-13 *	NDM	1024 (NWT)	16 (R)	64 (R)	64 (R)	1024 (R)	0.5 (S)	1 (S)
24-1-14 *	NDM+OXA-48	64 (WT)	256 (R)	64 (R)	0.5 (S)	1024 (R)	1 (S)	1 (S)
24-1-16	NDM+OXA-48	128 (WT)	256 (R)	32 (R)	0.5 (S)	1 (S)	0.5 (S)	0.0625 (S)
24-1-17	NDM	128 (WT)	2 (S)	64 (R)	0.5 (S)	1024 (R)	0.06 (S)	0.25 (S)
24-1-18	NDM	64 (WT)	4 (S)	64 (R)	0.5 (S)	1024 (R)	0.12 (S)	0.125 (S)
24-1-27 *	VIM-1	128 (WT)	16 (R)	64 (R)	0.5 (S)	1024 (R)	0.12 (S)	0.125 (S)
24-1-28 *	VIM-4	32 (WT)	16 (R)	16 (R)	0.5 (S)	64 (R)	0.12 (S)	0.5 (S)
24-1-29 *	VIM-20	32 (WT)	256 (R)	8 (I)	16 (R)	32 (R)	0.06 (S)	0.25 (S)
24-1-30 *	NDM-1	64 (WT)	32 (R)	64 (R)	0.5 (S)	1024 (R)	0.12 (S)	1 (S)
24-1-31 *	NDM-5	32 (WT)	32 (R)	64 (R)	0.5 (S)	1024 (R)	0.25 (S)	4 (R)
24-1-32	IMP-8	8 (WT)	8 (S)	1 (S)	0.5 (S)	256 (R)	0.25 (S)	8 (R)
24-1-39 *	NDM-1	32 (WT)	1 (S)	64 (R)	0.5 (S)	1024 (R)	0.12 (S)	16 (R)
24-1-40 *	NDM-1	8 (WT)	1 (S)	64 (R)	0.5 (S)	1024 (R)	0.06 (S)	16 (R)
24-1-41	NDM-1	64 (WT)	1 (S)	0.06 (S)	0.5 (S)	1 (S)	0.12 (S)	8 (R)
24-1-42	NDM-1	64 (WT)	4 (S)	64 (R)	0.5 (S)	1024 (R)	0.25 (S)	2 (S)
24-1-43 *	NDM-1	64 (WT)	16 (R)	64 (R)	0.5 (S)	1024 (R)	0.25 (S)	0.5 (S)
19-17-11 *	NDM-1+OXA-48	32 (WT)	256 (R)	64 (R)	64 (R)	1024 (R)	0.25 (S)	16 (R)

[a] MIC determined by agar dilution method. For FOS, the species-specific epidemiological cut-off (ECOFF; i.e., 128 mg/L) was used to distinguish between wild-type and non-wild-type isolates.
[b] MIC determined employing UMIC® microdilution.
* Multidrug-resistant (MDR) isolates, defined as non-susceptibility to at least one agent in three or more antimicrobial categories (see text); six antimicrobial categories were considered for the classification as MDR (modified from Magiorakos et al.[1]): (i) phosphonic acid derivatives (FOS), (ii) carbapenems (MER), (iii) beta-lactam/beta-lactamase inhibitors (CAZ-AVI, AZT-AVI), (iv) polymyxins (COL), (v) cephalosporins (FDC), and (vi) aminoglycosides (AMI).
The highlighted strain (24-1-18) was tested in the time-kill assay.
Abbreviations: *AMI* amikacin, *AZT-AVI* aztreonam-avibactam, *COL* colistin, *CTX-M* cefotaximase Munich, *CAZ-AVI* ceftazidime-avibactam, *ESBL* extended-spectrum beta-lactamase, *FDC* cefiderocol, *FOS* fosfomycin, *I* increased exposure (considered susceptible), *IMP* imipenemase, *MER* meropenem, *MIC* minimal inhibitory concentration, *NDM* New Delhi metallo-β-lactamase, *NWT* non-wild type (considered non-susceptible), *OXA* oxacillinase, *R* resistant, *S* susceptible, *TEM* Temoneira β-lactamase, *VIM* Verona-integron metallo-β-lactamase, *WT* wild type.

Table S2. Minimum inhibitory concentration (MIC) determination of carbapenemase-producing *P. aeruginosa* clinical isolates by reference method.

Strain	β-lactamase	MIC [mg/L] (category)						
		FOS [a]	MER	COL	AZT	CAZ-AVI	AZT-AVI [b]	FDC [c]
24-1-5 *	NDM	32 (WT)	64 (R)	1 (S)	32 (R)	1024 (R)	32	4 (R)
24-1-7	VIM	32 (WT)	0.5 (S)	2 (S)	8 (I)	2 (S)	16	0.06 (S)
24-1-9	VIM	4 (WT)	16 (R)	2 (S)	8 (I)	128 (R)	8	1 (S)
24-1-10 *	VIM	4 (WT)	64 (R)	16 (R)	16 (I)	32 (R)	16	0.25 (S)
24-1-23	VIM	64 (WT)	32 (R)	4 (S)	8 (I)	128 (R)	8	0.125 (S)
24-1-24	VIM	64 (WT)	32 (R)	1 (S)	16 (I)	128 (R)	16	0.5 (S)
24-1-25	IMP	2 (WT)	8 (I)	2 (S)	32 (R)	256 (R)	32	0.25 (S)
24-1-26 *	IMP	1024 (NWT)	64 (R)	2 (S)	64 (R)	128 (R)	64	2 (S)
24-1-33 *	NDM-1	64 (WT)	32 (R)	2 (S)	128 (R)	1024 (R)	16	4 (R)
24-1-34 *	VIM-4	32 (WT)	64 (R)	2 (S)	32 (R)	128 (R)	16	0.5 (S)
24-1-35	VIM-2	2 (WT)	64 (R)	4 (S)	8 (I)	128 (R)	8	0.25 (S)
24-1-36	VIM-2	16 (WT)	8 (I)	1 (S)	4 (I)	32 (R)	4	0.25 (S)
24-1-37 *	VIM-4	64 (WT)	64 (R)	1 (S)	32 (R)	512 (R)	32	2 (S)
24-1-38 *	IMP-7	32 (WT)	64 (R)	1 (S)	32 (R)	512 (R)	32	4 (R)
24-1-44	VIM	64 (WT)	32 (R)	1 (S)	8 (I)	1024 (R)	4	1 (S)
19-19-60	VIM-2	64 (WT)	64 (R)	1 (S)	8 (I)	32 (R)	8	0.5 (S)
19-47-41	VIM-2	16 (WT)	64 (R)	1 (S)	16 (I)	64 (R)	4	0.5 (S)
19-51-46	VIM-2	8 (WT)	64 (R)	2 (S)	8 (I)	32 (R)	16	0.125 (S)
19-69-27 *	IMP-12+OXA-35	1024 (NWT)	64 (R)	1 (S)	32 (R)	1 (S)	0.06	4 (R)
19-87-62	GIM-1	16 (WT)	64(R)	1 (S)	16 (I)	8 (S)	16	2 (S)

[a] MIC determined by agar dilution method. For FOS, the species-specific epidemiological cut-off (ECOFF; i.e., 256 mg/L) was used to distinguish between wild-type and non-wild-type isolates.
[b] No MIC breakpoint available.
[c] MIC determined employing UMIC® microdilution.
* Multidrug-resistant (MDR) isolates, defined as non-susceptibility to at least one agent in three or more antimicrobial categories (see text); six antimicrobial categories were considered for the classification as MDR (modified from Magiorakos et al. [1]): (i) phosphonic acid derivatives (FOS), (ii) carbapenems (MER), (iii) beta-lactam/beta-lactamase inhibitors (CAZ-AVI, AZT-AVI), (iv) polymyxins (COL), (v) cephalosporins (FDC), and (vi) monobactams (AZT).
The highlighted strain (24-1-44) was tested in the time-kill assay.
Abbreviations: *AZT* aztreonam, *AZT-AVI* aztreonam-avibactam, *COL* colistin, *CAZ-AVI* ceftazidime-avibactam, *FDC* cefiderocol, *FOS* fosfomycin, *GIM* German imipenemase, *I* increased exposure (considered susceptible), *IMP* imipenemase, *MIC* minimal inhibitory concentration, *MER* meropenem, *MIC* minimal inhibitory concentration, *NDM* New Delhi metallo-β-lactamase, *NWT* non-wild type (considered non-susceptible), *OXA* oxacillinase, *R* resistant, *S* susceptible, *VIM* Verona-integron metallo-β-lactamase, *WT* wild type.

Table S3. Susceptible breakpoint index (SBPI) of carbapenemase-producing *K. pneumoniae* clinical isolates from agar dilution checkerboard experiments.

	SPBI					
**Strain/antibiotic**	**FOS+MER**	**FOS+COL**	**FOS+AMI**	**FOS+CAZ-AVI**	**FOS+FDC**	**FOS+AZT-AVI**
24-1-1	4.25	2.13	10.00	64.50	8.25	64.50
24-1-2	4.25	6.00	16.00	65.00	66.00	66.00
24-1-3	16.06	2.25	8.50	2.50	0.75	129.00
24-1-6	16.13	8.13	12.00	65.00	10.00	40.00
24-1-12	0.14	2.13	8.00	4.25	0.75	8.25
24-1-13	2.03	2.25	6,00	4.25	2.25	64.50
24-1-14	4.50	6.00	8.50	67.67	36.00	66.00
24-1-16	1.00	4.00	4.06	513.00	40.00	66.00
24-1-17	8.50	9.00	16.50	65.00	68.00	260.00
24-1-18	16.00	3.00	4.13	65.00	70.67	260.00
24-1-27	2.06	3.00	48.00	65.00	10.00	257.00
24-1-28	16.00	10.00	66.00	65.00	16.00	66.00
24-1-29	132.00	16.13	8.06	65.00	40.00	64.00
24-1-30	16.25	9.00	130.00	65.00	6.00	132.00
24-1-31	2.13	4.50	2.50	33.00	10.00	68.00
24-1-32	136.00	8194.00	16.00	65.00	68.00	192.00
24-1-39	16.25	8194.00	0.19	65.00	34.00	320.00
24-1-40	64.25	8194.00	17.00	65.00	33.00	64.00
24-1-41	32.25	5.00	4.25	514.00	36.00	4298.67
24-1-42	8.13	3.00	0.19	65.00	17.00	132.00
24-1-43	16.13	4.50	128.50	65.00	12.00	130.00
19-17-11	8.06	4.13	4.50	65.00	1.25	130.00
**Range**	**0.14-136.00**	**2.13-8194.00**	**0.19-130.00**	**2.50-514.00**	**0.75-70.67**	**8.25-4298.67**
**Mean (SD)**	**23.74 (37.40)**	**1122.10 (2810.09)**	**24.33 (37.34)**	**96.05 (133.72)**	**26.63 (23.48)**	**312.63 (873.72)**
**Median (IQR)**	**12.25 (4.25-16.22)**	**4.75 (3.00-9.00)**	**8.50 (4.25-16.50)**	**65.00 (65.00-65.00)**	**16.50 (8.69-39.00)**	**98.50 (64.88-177.00)**

A SBPI ≥ 2 indicates that the MICs of combined antimicrobials are equal or lower than their respective susceptibility breakpoints (or ECOFFs in case of FOS). Thus, the greater the SBPI value, the more effective the antimicrobial combination [2,3].
The highlighted strain (24-1-18) was tested in the time-kill assay.
Abbreviations: *AMI* amikacin, *AZT-AVI* aztreonam-avibactam, *COL* colistin, *CAZ-AVI* ceftazidime-avibactam, *ECOFF* epidemiological cut-off, *FDC* cefiderocol, *FOS* fosfomycin, *IQR* interquartile range, *MER* meropenem, *SD* standard deviation, *SPBI* susceptible breakpoint index.

Table S4. Susceptible breakpoint index (SBPI) of carbapenemase-producing *P. aeruginosa* clinical isolates from agar dilution checkerboard experiments.

	SBPI					
**Strain/antibiotic**	**FOS+MER**	**FOS+COL**	**FOS+AZT**	**FOS+CAZ-AVI**	**FOS+FDC**	**FOS+AZT-AVI [a]**
24-1-5	32.25	96.00	34.00	72.00	17.00	NA
24-1-7	80.00	80.00	32.00	34.00	1088.00	NA
24-1-9	129.00	320.00	520.00	128.00	576.00	NA
24-1-10	512.25	1028.00	260.00	160.00	2080.00	NA
24-1-23	40.00	36.00	20.00	68.00	2080.00	NA
24-1-24	18.00	80.00	12.00	40.00	2064.00	NA
24-1-25	264.00	320.00	516.00	128.00	2112.00	NA
24-1-26	0.50	2.25	1.25	0.56	1.00	NA
24-1-33	16.25	80.00	4.13	68.00	18.00	NA
24-1-34	32.50	80.00	32.50	80.00	80.00	NA
24-1-35	512.06	320.00	288.00	80.00	1088.00	NA
24-1-36	34.00	272.00	260.00	80.00	2112.00	NA
24-1-37	16.13	144.00	10.00	68.00	24.00	NA
24-1-38	64.50	136.00	9.00	10.00	32.00	NA
24-1-44	36.00	80.00	24.00	68.00	34.00	NA
19-19-60	16.50	80.00	24.00	68.00	1088.00	NA
19-47-41	33.00	288.00	65.00	80.00	1088.00	NA
19-51-46	16.50	136.00	96.00	32.00	64.00	NA
19-69-27	0.31	80.00	1.25	0.56	4.50	NA
19-87-62	33.00	272.00	6.00	72.00	64.00	NA
**Range**	**0.31-512.25**	**2.25-1028.00**	**1.25-516.00**	**0.56-160.00**	**1.00-2112.00**	**-**
**Mean (SD)**	**94.34 (150.54)**	**196.51 (216.29)**	**110.76 (162.49)**	**66.86 (39.96)**	**785.73 (853.56)**	**-**
**Median (IQR)**	**33.00 (16.50-68.38)**	**116.00 (80.00-270.00)**	**28.00 (9.75-137.00)**	**68.00 (38.50-80.00)**	**328.00 (30.00-1332.00)**	**-**

A SBPI ≥ 2 indicates that the MICs of combined antimicrobials are equal or lower than their respective susceptibility breakpoints (or ECOFFs in case of FOS). Thus, the greater the SBPI value, the more effective the antimicrobial combination [2,3].
The highlighted strain (24-1-44) was tested in the time-kill assay.
[a] For AZT-AVI, EUCAST does not provide a clinical breakpoint for *P. aeruginosa.*
Abbreviations: *AZT* aztreonam, *AZT-AVI* aztreonam-avibactam, *COL* colistin, *CAZ-AVI* ceftazidime-avibactam, *ECOFF* epidemiological cut-off, *FDC* cefiderocol, *FOS* fosfomycin, *IQR* interquartile range, *MER* meropenem, *NA* not available, *SD* standard deviation, *SPBI* susceptible breakpoint index.

Table S5: Individual fractional inhibitory concentration (FIC) values of the FOS+CAZ-AVI combination against MBL-producing *K. pneumoniae* clinical isolates.

Strain	β-lactamase(s)	Individual FIC	
		$FIC_{CAZ\text{-}AVI \rightarrow FOS}$	$FIC_{FOS \rightarrow CAZ\text{-}AVI}$
24-1-1	VIM+ESBL	**0.250**	**0.001**
24-1-2	NDM+ESBL	2.000	**0.001**
24-1-3	VIM+TEM	**0.250**	**0.031**
24-1-6	NDM+VIM+CTX-M+TEM	**0.500**	**0.001**
24-1-12	NDM+CTX-M+TEM	1.000	**0.016**
24-1-13	NDM	1.000	**0.016**
24-1-14	NDM+OXA-48	**0.500**	**0.001**
24-1-16	NDM+OXA-48	**0.0005**	**0.063**
24-1-17	NDM	1.000	**0.001**
24-1-18	NDM	**0.250**	**0.001**
24-1-27	VIM-1	**0.250**	**0.001**
24-1-28	VIM-4	**0.500**	**0.001**
24-1-29	VIM-20	**0.250**	**0.001**
24-1-30	NDM-1	**0.500**	**0.001**
24-1-31	NDM-5	**0.250**	**0.002**
24-1-32	IMP-8	2.000	**0.001**
24-1-39	NDM-1	**0.500**	**0.001**
24-1-40	NDM-1	**0.500**	**0.001**
24-1-41	NDM-1	**0.001**	**0.125**
24-1-42	NDM-1	**0.500**	**0.001**
24-1-43	NDM-1	**0.500**	**0.001**
19-17-11	NDM-1+OXA-48	32.000	**0.001**

Greyed fields indicate a synergistic effect of either CAZ-AVI on FOS ($FIC_{CAZ\text{-}AVI \rightarrow FOS}$) or of FOS on CAZ-AVI ($FIC_{FOS \rightarrow CAZ\text{-}AVI}$) against *K. pneumoniae* clinical isolates (FIC ≤ 0.25). Bold values indicate an additive effect
(0.25 < FIC ≤ 0.5). The highlighted strain (24-1-18) was tested in the time-kill assay. Abbreviations: *CTX-M* cefotaximase Munich, *CAZ-AVI* ceftazidime-avibactam, *ESBL* extended-spectrum β-lactamase, *FIC* fractional inhibitory concentration, *FOS* fosfomycin, *IMP* imipenemase, *NDM* New Delhi metallo-β-lactamase*, OXA* oxacillinase, *TEM* Temoneira β-lactamase, *VIM* Verona-integron metallo-β-lactamase.

Table S6: Individual fractional inhibitory concentration (FIC) values of the FOS+CAZ-AVI combination against MBL-producing *P. aeruginosa* clinical isolates.

Strain	β-lactamase(s)	Individual FIC	
		$FIC_{CAZ\text{-}AVI \rightarrow FOS}$	$FIC_{FOS \rightarrow CAZ\text{-}AVI}$
24-1-5	NDM	1.000	**0.001**
24-1-7	VIM	**0.250**	**0.250**
24-1-9	VIM	2.000	**0.008**
24-1-10	VIM	**0.500**	**0.002**
24-1-23	VIM	**0.500**	**0.001**
24-1-24	VIM	**0.500**	**0.002**
24-1-25	IMP	1.000	**0.001**
24-1-26	IMP	0.500	1.000
24-1-33	NDM-1	1.000	**0.001**
24-1-34	VIM-4	**0.500**	**0.001**
24-1-35	VIM-2	1.000	**0.004**
24-1-36	VIM-2	**0.500**	**0.001**
24-1-37	VIM-4	1.000	**0.001**
24-1-38	IMP-7	1.000	**0.031**
24-1-44	VIM	1.000	**0.001**
19-19-60	VIM-2	**0.500**	**0.001**
19-47-41	VIM-2	**0.500**	**0.001**
19-51-46	VIM-2	2.000	**0.004**
19-69-27	IMP-12+OXA-35	**0.500**	1.000
19-87-62	GIM-1	1.000	**0.001**

Greyed fields indicate a synergistic effect of either CAZ-AVI on FOS ($FIC_{CAZ\text{-}AVI \rightarrow FOS}$) or of FOS on CAZ-AVI ($FIC_{FOS \rightarrow CAZ\text{-}AVI}$) against *P. aeruginosa* clinical isolates (FIC ≤ 0.25). Bold values indicate an additive effect
(0.25 < FIC ≤ 0.5). The highlighted strain (24-1-44) was tested in the time-kill assay. Abbreviations: *CAZ-AVI* ceftazidime-avibactam, *FIC* fractional inhibitory concentration, *FOS* fosfomycin, *GIM* German imipenemase, *IMP* imipenemase, *NDM* New Delhi metallo-β-lactamase, *OXA* oxacillinase, *VIM* Verona-integron metallo-β-lactamase.

References

1. Magiorakos, A.-P.; Srinivasan, A.; Carey, R.B.; Carmeli, Y.; Falagas, M.E.; Giske, C.G.; Harbarth, S.; Hindler, J.F.; Kahlmeter, G.; Olsson-Liljequist, B.; et al. Multidrug-Resistant, Extensively Drug-Resistant and Pandrug-Resistant Bacteria: An International Expert Proposal for Interim Standard Definitions for Acquired Resistance. *Clin Microbiol Infect* **2012**, *18*, 268–281, doi:10.1111/j.1469-0691.2011.03570.x.

2. Nussbaumer-Pröll, A.; Obermüller, M.; Weiss-Tessbach, M.; Eberl, S.; Zeitlinger, M.; Matiba, B.; Mayer, C.; Kussmann, M. Synergistic Activity of Fosfomycin and Flucloxacillin against Methicillin-Susceptible and Methicillin-Resistant Staphylococcus Aureus: In Vitro and in Vivo Assessment. *Med Microbiol Immunol* **2025**, *214*, 32, doi:10.1007/s00430-025-00841-3.

3. Milne, K.E.N.; Gould, I.M. Combination Testing of Multidrug-Resistant Cystic Fibrosis Isolates of Pseudomonas Aeruginosa: Use of a New Parameter, the Susceptible Breakpoint Index. *J Antimicrob Chemother* **2010**, *65*, 82–90, doi:10.1093/jac/dkp384.
